# A lightweight network based on dual-stream feature fusion and dual-domain attention for white blood cells segmentation

**DOI:** 10.3389/fonc.2023.1223353

**Published:** 2023-09-04

**Authors:** Yang Luo, Yingwei Wang, Yongda Zhao, Wei Guan, Hanfeng Shi, Chong Fu, Hongyang Jiang

**Affiliations:** ^1^ School of Mathematics and Information Science, Anshan Normal University, Anshan, Liaoning, China; ^2^ School of Applied Technology, Anshan Normal University, Anshan, Liaoning, China; ^3^ Department of Computer Science and Engineering, Southern University of Science and Technology, Shenzhen, Guangdong, China; ^4^ School of Computer Science and Engineering, Northeastern University, Shenyang, China; ^5^ Engineering Research Center of Security Technology of Complex Network System, Ministry of Education, Shenyang, China; ^6^ Key Laboratory of Intelligent Computing in Medical Image, Ministry of Education, Northeastern University, Shenyang, China

**Keywords:** white blood cells segmentation, instance segmentation, YOLACT-CIS, dual-stream feature fusion network (DFFN), dual-domain attention module (DDAM)

## Abstract

**Introduction:**

Accurate white blood cells segmentation from cytopathological images is crucial for evaluating leukemia. However, segmentation is difficult in clinical practice. Given the very large numbers of cytopathological images to be processed, diagnosis becomes cumbersome and time consuming, and diagnostic accuracy is also closely related to experts' experience, fatigue and mood and so on. Besides, fully automatic white blood cells segmentation is challenging for several reasons. There exists cell deformation, blurred cell boundaries, and cell color differences, cells overlapping or adhesion.

**Methods:**

The proposed method improves the feature representation capability of the network while reducing parameters and computational redundancy by utilizing the feature reuse of Ghost module to reconstruct a lightweight backbone network. Additionally, a dual-stream feature fusion network (DFFN) based on the feature pyramid network is designed to enhance detailed information acquisition. Furthermore, a dual-domain attention module (DDAM) is developed to extract global features from both frequency and spatial domains simultaneously, resulting in better cell segmentation performance.

**Results:**

Experimental results on ALL-IDB and BCCD datasets demonstrate that our method outperforms existing instance segmentation networks such as Mask R-CNN, PointRend, MS R-CNN, SOLOv2, and YOLACT with an average precision (AP) of 87.41%, while significantly reducing parameters and computational cost.

**Discussion:**

Our method is significantly better than the current state-of-the-art single-stage methods in terms of both the number of parameters and FLOPs, and our method has the best performance among all compared methods. However, the performance of our method is still lower than the two-stage instance segmentation algorithms. in future work, how to design a more lightweight network model while ensuring a good accuracy will become an important problem.

## 1 Introduction

Blood Cancer is a major killer worldwide. Leukemia is the most common blood cancer and a liquid malignancy (1). Among the top 10 cancer deaths in China, about 60000 people died of leukemia every year (2).

Early diagnosis of leukemia can greatly improve the survival rate. The early diagnosis of leukemia is usually made by doctors observing the morphology and structure of bone marrow and blood cells under a microscope, such as microscopic examination of bone marrow aspiration and blood smears (3). Given the very large numbers of cytopathological images to be processed, diagnosis becomes cumbersome and time consuming for doctors, and diagnostic accuracy is also closely related to experts’ experience, fatigue and mood and so on. In view of the facts many researchers have proposed some methods (4–13) for diagnosis of leukemia. The critical step of which is segmentation. Thus, there is an increasing requirement for a reproducible fully automatic white blood cells segmentation method to accelerate and ease the process of diagnosis, therapy and treatment.

Fully automatic white blood cells segmentation is challenging for several reasons. First, cytopathological image datasets are usually collected by hospitals through different equipment under different lighting and staining conditions. Second, the influence of human in the process of making cell smears or slices leads to occurrences of cell deformation, blurred cell boundaries, and cell color differences. Third, in a cytopathological image, there are many cells and the shape and structure of cells are complex, which makes the size and shape of different cells vary greatly, leading to some cells overlapping or adhesion (14–16). Nowadays, white blood cells segmentation is still an open problem, attracting much interest and stimulating the further development of automatic segmentation methods. Up to now, a wide range of cell segmentation methods has been proposed, including region growing methods (17), hough transform methods (18), filtering methods (19), thresholding methods (20–24), watershed methods (25, 26), clustering methods (27–29), SVM methods (30, 31), edge methods (32–35) and other methods (36, 37). Although the above traditional segmentation methods have achieved acceptable results, there are still some limitations and challenges. Because the understanding and analysis of complex images usually requires high-level semantic information, the traditional segmentation methods need hand-crafted feature extraction, and can only extract low-level information. In light of the complex cell morphology, these methods have poor robustness, especially for the cell adhesion and blurred cell boundaries they have poor segmentation ability.

In recent years, the performance of convolutional neural networks (CNNs) in the ImageNet large scale visual recognition challenge (38) has merited the description *state-of-the-art*. Shelhamer et al. (39) substituted the convolution layer for the fully connected layer of CNN, and thus constructed a fully convolutional network (FCN) to achieve automatic semantic segmentation of images. Based on FCN, Ronneberger et al. (40) proposed a U-net, using the idea of spanning connection, which enabled the network to acquire information from both shallow and deep layers at the same time. Compared with traditional methods, in which segmentation is based on manually identified features, CNNs can automatically extract the most intricate semantic features resulting in improved white blood cells segmentation (41–43).

In 2018, Tran et al. (44) used SegNet (45) to achieve cell segmentation in blood smears, but overlapping cells could not be effectively separated. Guerrero-Pena et al. (14) proposed a multi-class weighted loss function for cell instance segmentation. The loss function was used to adjust the category imbalance, and thus the cell contour was focused on. By increasing the weight of adhesive cell boundaries, the network can more accurately capture the adhesive boundaries. Schmidt et al. (15) proposed a STARDIST. According to characteristics of cell shape that are similar to a circle, they used polygons to detect and segment cells. This method showed excellent performance in dealing with the dense cell adhesion problem. In 2019, Daniel et al. (46) proposed a single stage instance algorithm YOLACT, which multiplied the prototypes and the mask generated by the semantic segmentation network to produce instance masks. Therefore, the YOLACT had extremely fast speed and can meet the requirements of real-time segmentation. In 2019, Fan et al. (47) proposed a LeukocyteMask method, which first located the white blood cell regions, and then segmented white blood cells in the regions. This method can avoid background interference, and improve the network performance. Yi et al. (48) combined the object detection network SSD and U-net to segment cells, and achieved excellent results for neural cell instance segmentation. Graham et al. (16) proposed a new CNN Hover-net for synchronous nuclear segmentation and classification, which trained the vertical and horizontal distances feature information of nuclei to attain a distance weight map, and then the distance weight map was post-processed through the watershed method. This network provided a good idea of segmentation for solving the problem of clustered nuclei. In 2020, Zhou et al. (49) proposed a novel deep semi-supervised knowledge distillation framework, called MMT-PSM, for overlapping cervical cell instance segmentation. To solve the problem of low medical image data, both labeled and unlabeled image data were used to train the segmentation network, and the segmentation accuracy through knowledge distillation was improved. In 2021, Xie et al. (50) proposed a popularmask++ instance segmentation model, which transformed the instance segmentation problem into predicting contours of objects in polar coordinate, and unified instance segmentation and object detection into one framework by using coordinate representation. In 2022, Chan et al. (51) proposed an encoding-decoding network with Res2-UneXt, which included a simple and effective data augmentation method. In 2023, Dhalls et al. (52) proposed an encoder–decoder model based on deep learning to focus on salient multiscale features of white blood cells, which combined features extracted from standard and dilated convolutions. Zhou et al. (53) proposed a novel dual-task framework, which used a novel color activation mapping block to produce a refined salient map as the final salient map, and then a novel adaptive threshold strategy was proposed to automatically segment the white blood cells from the final salient map. Abrol et al. (54) proposed a white blood cells segmentation method in which three color spaces are considered for image augmentation. The proposed algorithm uses a marker-based watershed algorithm and peak local maxima.

Althought the cell segmentation methods based on deep learning have achieved much more results than traditional methods, there are also the following deficiencies in cell segmentation research:

(1) There is a relationship between the morphological characteristics of cells and the types of diseases, and when making cytopathological images, there are often cell adhesion (14–16). Therefore, how to segment adhesive cells is a research difficulty. Nowadays researchers segment cells by the semantic segmentation methods, and then extract cell contours through post-processing, but the segmentation effect of cell contours is still poor.
(2) Most of the existing segmentation networks are proposed for natural images, and no distinctive designs are made for the characteristics of cytopathological images. It is worth noting that the existing instance segmentation networks for cytopathological images are often complex and redundant, which makes the network model difficult to apply in clinical practice.

Motivated by above problems, according to the characteristics of cells in cytopathology images, to realize white blood cell detection and segmentation in cytopathology images, a cytopathology image instance segmentation model named YOLACT-CIS based on the instance segmentation frame YOLACT is proposed. The experimental results demonstrate that our method outperforms the existing methods.

Our study makes the following contributions:

(1) Taking the advantage of feature reuse of Ghost module, the single-stage instance segmentation algorithm YOLACT is redesigned to reconstruct the backbone network, aiming at making the backbone network lightweight, thereby reducing the number of the network parameters and computational complexity.
(2) The feature fusion layer in the instance segmentation algorithm for white blood cells is redesigned, and a dual-stream feature fusion network (DFFN) is proposed, which enhances the flow of information from shallow layers by adding an extra bottom-up fusion path in the feature pyramid, thereby improving the segmentation effect of adhesive cells and blurred cell boundaries.
(3) A dual-domain attention module (DDAM) is designed to extract global features from both frequency and spatial domains simultaneously. The feature information obtained from two different domains is complementary to each other, thereby enhancing extraction of cell details and improving the segmentation effect of adhesive cells and blurred cell boundaries.

The rest of this paper is organized as follows. Section II presents the proposed method. Section III provides the experimental details and results. The discussion is presented in Section IV. Finally, Section V offers some conclusions.

## 2 Methods

### 2.1 Instance segmentation

At present, most of the existing instance segmentation algorithms (46, 55–57) are proposed for natural images, which can detect and segment objects at the same time. For cell segmentation tasks, instance segmentation algorithms can often achieve better results than semantic segmentation algorithms when dealing with cell adhesion. Nowadays, the instance segmentation algorithms are mainly divided into single-stage instance segmentation algorithms and two-stage instance segmentation algorithms. The detection and segmentation in the two-stage instance segmentation algorithms are carried out step by step, which can get better segmentation accuracy, but usually have higher computational complexity and slower reasoning speed. The single-stage instance segmentation algorithms perform detection and segmentation tasks simultaneously in the network. In most cases, compared with the two-stage instance segmentation algorithms, the segmentation accuracy of the single-stage instance segmentation algorithms has a certain decline, but they can attain faster reasoning speed. The YOLACT method belongs to the single-stage instance segmentation algorithms. Although its segmentation accuracy is slightly reduced compared with the two-stages instance segmentation algorithms, it can achieve a good balance between accuracy and speed when dealing with downstream tasks such as cell segmentation.

**Figure 1:**
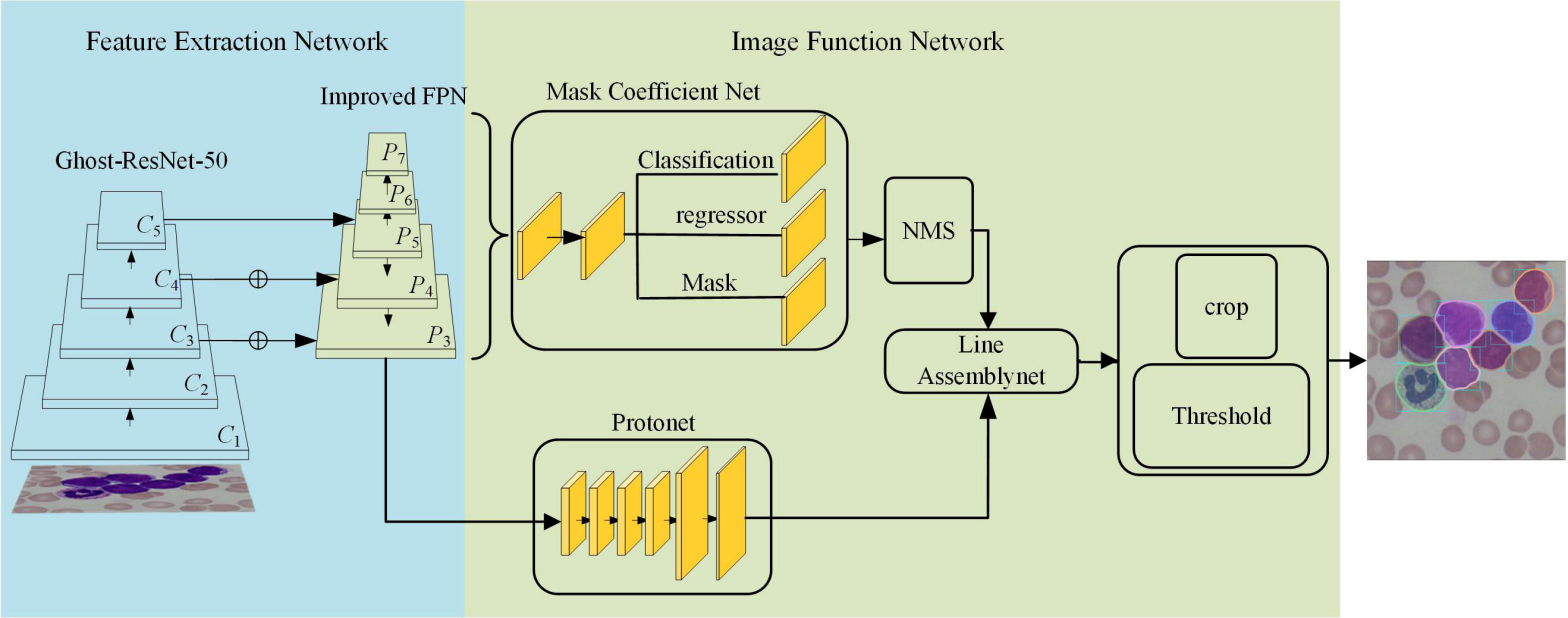
Schematic diagram of YOLACT-CIS.

This paper uses the YOLACT method as the basic architecture, and proposes a cytopathology image instance segmentation model named YOLACT-CIS (YOLACT-Cell Instance Segmentation) network to realize white blood cells segmentation in cytopathology images, as shown in **Figure 1**. The blue part is composed of the backbone network (Ghost-ResNet (58, 59) except fully connected layer) and the improved feature pyramid, which is mainly used for feature extraction. The green part mainly consists of mask coefficient network and prototype network, and is used to generate instance mask. The cell segmentation process is the following: First, the backbone network is used to primarily extract features of the input images, following which, the improved feature pyramid structure is used to further encode the extracted features at different stages. Second, the features of *P*_3_-*P*_7_ and *P*_3_ layers are fed into the mask coefficient network and the prototype network, respectively. At the same time, a series of coefficients generated by the mask coefficient network are multiplied with the mask generated by the prototype network to obtain the instance mask. Finally, the final results are attained through cropping the prediction box.

### 2.2 YOLACT-CIS network

#### 2.2.1 Lightweight backbone network

**Figure 2:**
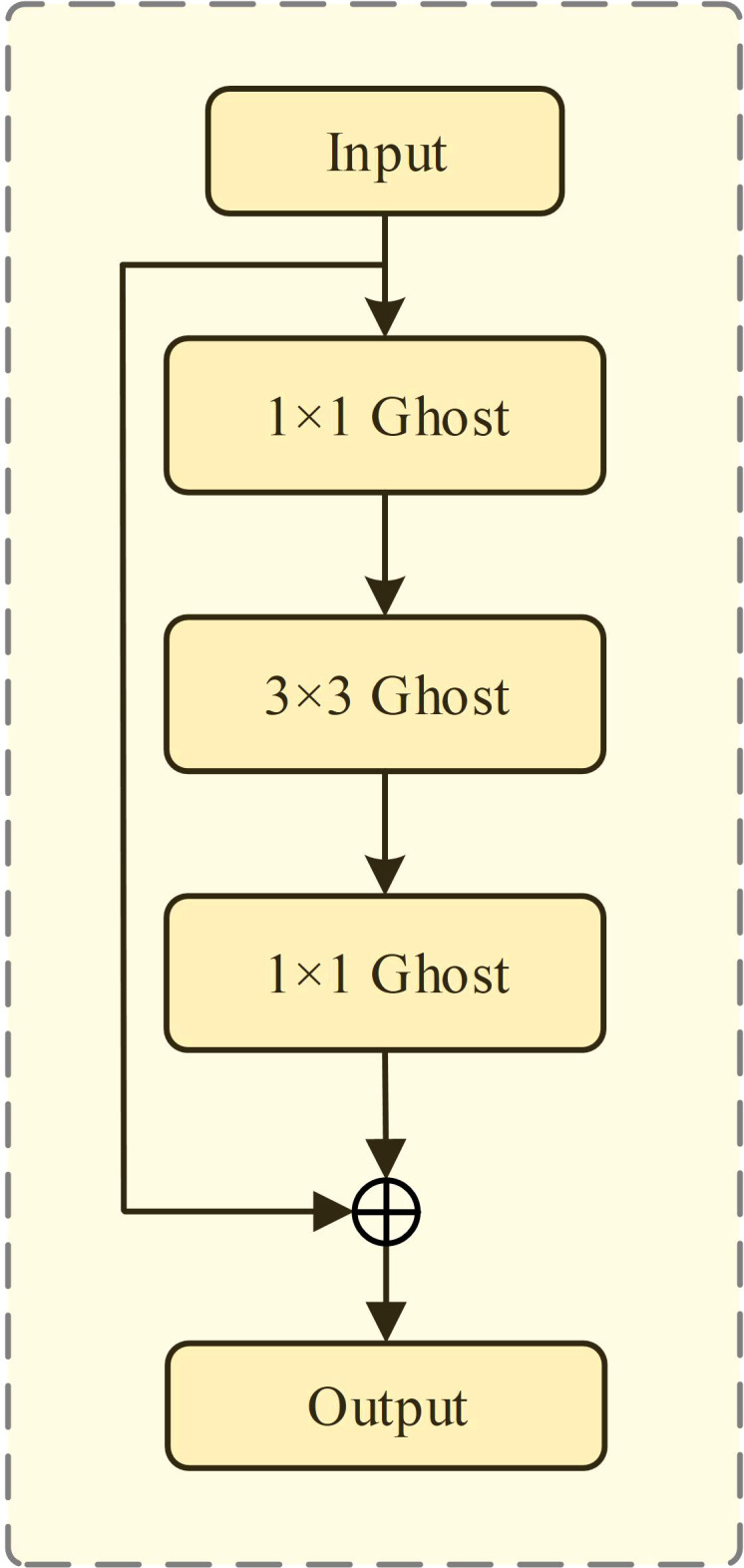
Schematic diagram of Ghost-ResNet.

According to the imaging characteristics of white blood cells in blood smear images, the morphological characteristics and color of white blood cells are obviously different from the surrounding background, instead white blood cells are very similar to each other. Therefore, if the network has too many parameters and is too complex, it may lead to the network over-fitting, thereby resulting in low network utilization, parameter redundancy and other problems. In order to resolve the aforementioned problems, making the backbone network lightweight is needed by reducing the amount of parameters in our method. In addition, with the deepening of the backbone network, the number of parameters and the amount of computation increase rapidly, in order to make our network easily deployed to practical applications, the lightweight backbone network are also needed. In GhostNet (59), a more efficient and lightweight convolution is proposed, which allows similar feature transfomations to be applied to redundant features, thereby reducing computational overhead. Inspired by this, in this paper, the standard convolution in the residual module of the backbone network is replaced with the Ghost module, as shown in **Figure 2**.

#### 2.2.2 Feature fusion layer

When using the deep convolutional networks to extract image features, the deep layers contain more high-level semantic features, and the shallow layers contain a lot of detailed information, such as positioning information. The YOLACT also uses the feature fusion network structure based on the feature pyramid, but only uses the feature layers of the last three stages of the backbone network, instead of the feature layer (*C*_1_) of the first stage and the the feature layer (*C*_2_) of the second stage in the top-down feature fusion. The reason is that when the *C*_1_ and *C*_2_ layers conduct the top-down feature fusion, the network performance is not significantly improved, on the contrary, the computational cost is increased. The information from the shallow layers is very important for object positioning and segmentation. Therefore, the feature pyramid is improved by adding a bottom-up path to enhance the flow of information from the shallow layers.

The feature pyramid network (FPN) (60) combines the features from the shallow and deep layers, thereby completing the multi-scale object detection task with less computational cost. In spite of the FPN structure in the YOLACT can better combine the information from the shallow and deep layers to improve the network performance, there still exists a problem of insufficient utilization of the information from the shallow layers.

**Figure 3:**
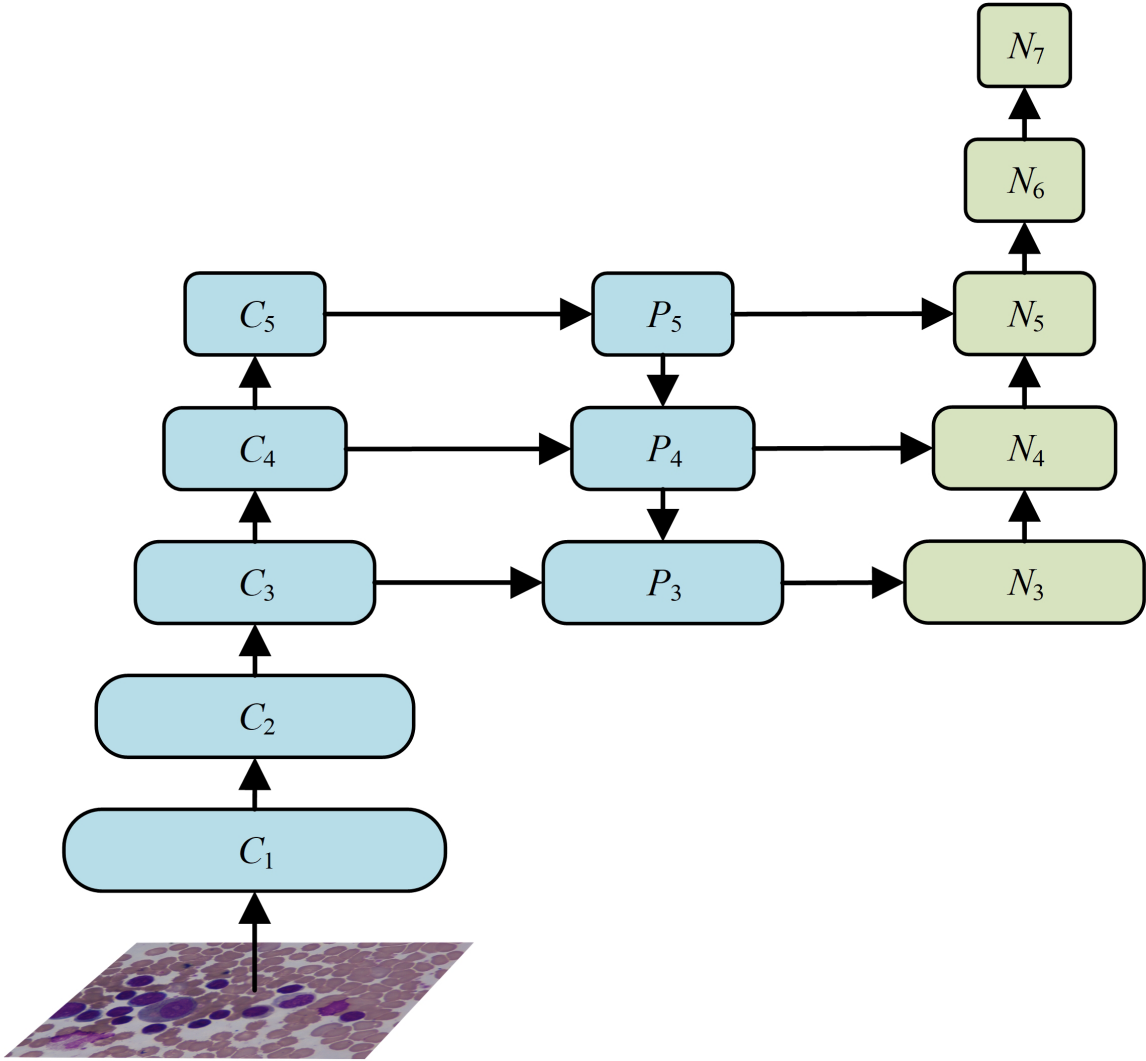
The structure of DFFN.

It is worth noting that the deep layers contain less detailed features, leading to the lack of positioning information from the shallow layers in the deep layers, as a result, white blood cells segmentation is not accurate enough. In view of the above-mentioned facts, according to the characteristics of white blood cells, a dual-stream feature fusion networks (DFFN) is proposed, as shown in **Figure 3**.

**Figure 4:**
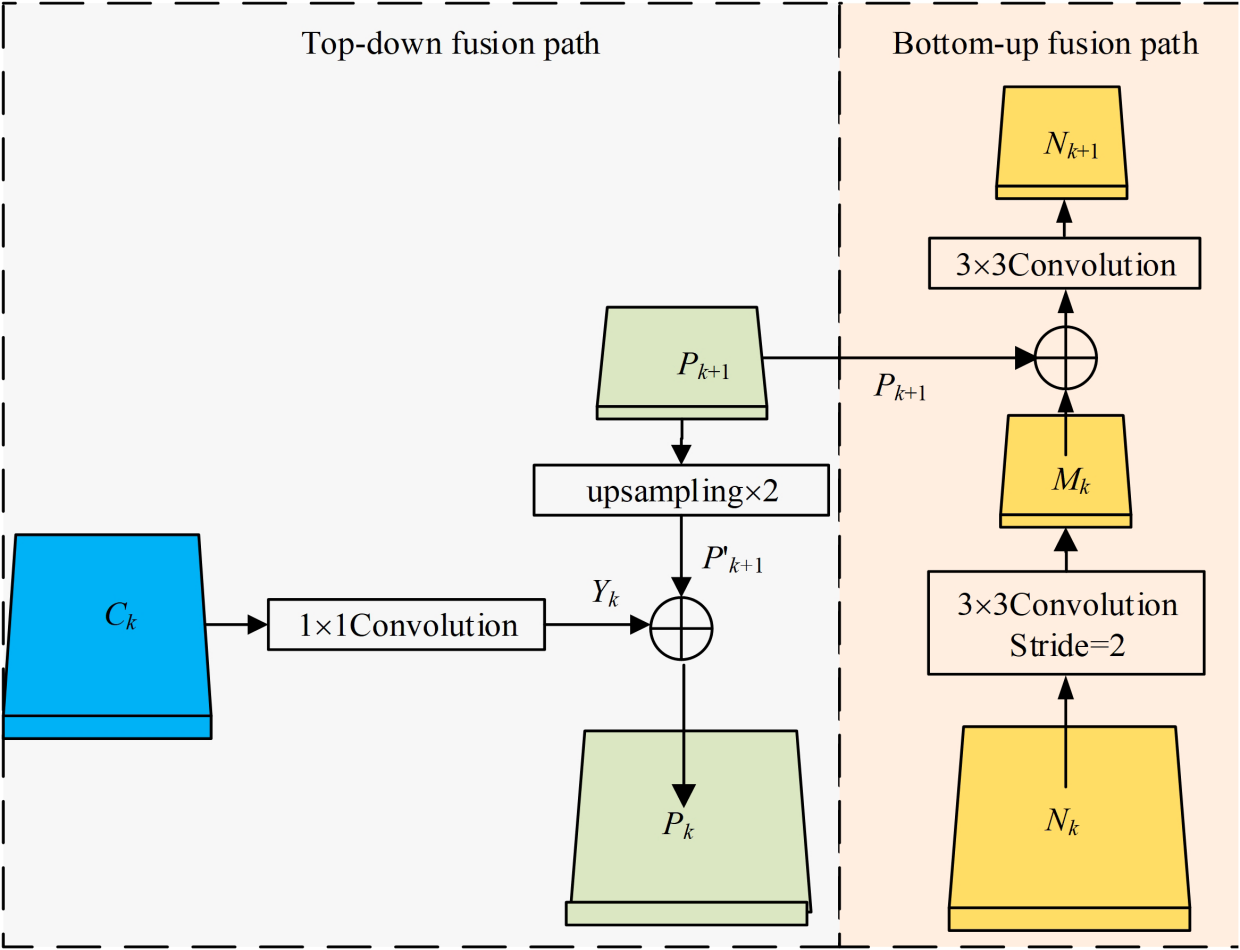
Feature fusion calculation process.

The DFFN combines the ideas of PANet (61) and FPN, and it can better transmit the detailed features, such as positioning and edge information, from the shallow layers to the deep layers through a bottom-up transmission. This can effectively promote the information flow of the shallow layers, and through information fusion for the shallow and deep layers, the DFFN can better obtain the detailed information of cells. Therefore, the DFFN can effectively improve accuracy of cell detection and segmentation. The fusion calculation process of the DFFN is shown in **Figure 4**.

The process of the top-down feature fusion of the DFFN: First, the feature map from the last layer *C_k_* is fed into a 1×1 convolution to generate the feature map *Y_k_*. Second, the feature map *P_k_*_+1_ is enlarged twice by upsampling to obtain the feature map *P* ^′^ *_k_*_+1_, which has the same dimension as the feature map *Y_k_*. Finally, *P_k_* is obtained by adding the feature map *Y_k_* and the feature map *P* ^′^ *_k_*_+1_, where *C*_5_ is fed into a 1×1 convolution to generate *P*_5_. In addition, *P*_5_, *P*_4_ and *P*_3_ all have 256 channels.

The process of the bottom-up feature fusion: First, feature map *N_k_* from the shallow layers is downsampled twice by a convolution to obtain the feature map *M_k_* of the same size as *P_k+_*_1._ Second, pixelwise addition of *P_k+_*_1_ and *M_k_* is performed, followed by a 3 × 3 convolution, thereby better achieving the feature fusion. Finally, *N_k_*_+1_ is obtained, where all *N*_5_, *N*_4_, and *N*_3_ have 256 channels. *N*_6_ and *N*_7_ are obtained from *N*_5_ and *N*_7_ by downsampling, respectively.

#### 2.2.3 Dual-domain attention module

**Figure 5:**
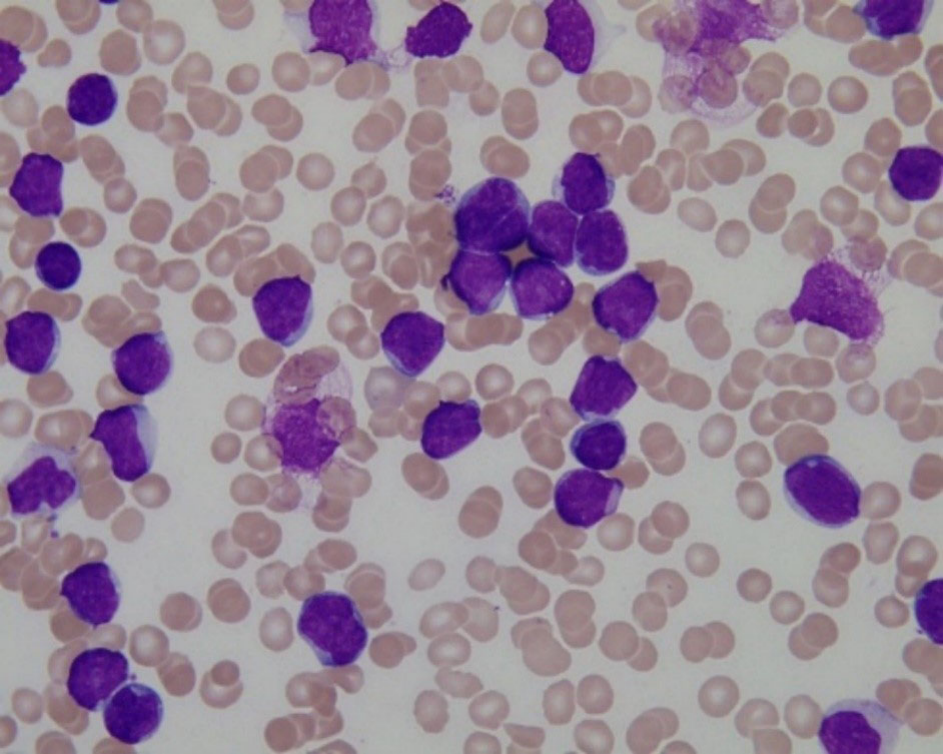
Cytopathological image.

There are not only white blood cells but also other cells in cytopathological images. In addition, the process of making blood smear may be affected by human and machines, which will reduce the imaging quality of blood smear. These factors will have a great impact on the accurate white blood cells segmentation. In this paper, channel attention can be used to focus on feature information of white blood cells in the channel domain, which makes the feature information of white blood cells easier extracted. Additionally, it can be seen from **Figure 5** that there exists not only adhesion between white blood cells, but also similarity, both of which contribute to the indistinct white blood cell boundaries. Using spatial attention mechanism can make a network pay attention to the details of white blood cell boundaries in the spatial domain, thus effectively distinguishing white blood cell boundaries.

In recent years, Attention mechanism has drawn much attention and shown promising results in medical image segmentation. As a representative of channel attention mechanism, SENet (62) recalibrates channels according to the importance of each channel. Convolutional block attention module (CBAM) (63), on the basis of SENet, increases its attention to spatial association. In this paper, based on CBAM, a spectrum based hybrid attention mechanism is proposed to enhance the attention to cell details.

CBAM module models the correlation between channel and spatial information from feature maps, thereby making the network focus on the key information from feature maps and improving representation capability of the network. This module calculates the attention distribution of feature maps from the channel and spatial domains, respectively. In the channel attention module of CBAM, it extracts key information by compressing spatial information. Compared to SENet using only one pooling strategy to extract feature information, both global max pooling (GMP) and global average pooling (GAP) are used in CBAM, which can comprehensively extract feature information, so that the network can obtain better performance. Although CBAM can effectively improve the network performance by using two pooling methods, there is still a problem of losing some key information from feature maps. Accordingly, more feature extraction methods are used to extract effective information from multiple aspects, aiming at improving the key information extraction capability of the network for objects. The channel attention mechanism proposed in this paper is different from the global feature extraction method in CBAM. It can extract features from the frequency domain, aiming at attaining more comprehensive features.

From the perspective of frequency domain, GAP is a special case of frequency components, that is, when only GAP is used to extract features, the information contained in other frequency components is not fully utilized. In order to resolve this problem, two-dimensional discrete cosine transform (2D-DCT) (64) is employed in our scheme, 2D-DCT of each channel from feature maps is defined by:


(1) $$Y_{h,w}^{k}=\sum_{h=0}^{H-1}\sum_{w=0}^{W-1}F_{k}\left(i,j\right)\cos\left(\frac{\pi h}{H}\left(i+\frac{1}{2}\right)\right)\cos\left(\frac{\pi w}{W}\left(j+\frac{1}{2}\right)\right)$$

$$\begin{array}{l} \text{ s.t. }i\in \left\{0,1,\cdots ,H-1\right\},j\in \left\{0,1,\cdots ,W-1\right\},\;h\in \left\{0,1,\cdots ,H-1\right\},\\ w\in \left\{0,1,\cdots ,W-1\right\},k\in \left\{0,1,\cdots ,C\right\} \end{array}$$

where $Y_{h,w}^{k}\in R^{C\times H\times W}$ is a 2D-DCT of a channel, and .. is the position (*i*, *j*) of the *k*-th channel from the feature map. $Y_{0,0}^{k}$ is the lowest frequency component of . $Y_{h,w}^{k}\in R^{C\times H\times W}$ . and defined as:


(2) $$\begin{array}{c} Y_{0,0}^{k}=\sum_{h=0}^{H-1}\sum_{w=0}^{W-1}f_{k}\left(i,j\right)\cos\left(\frac{\pi 0}{H}\left(i+\frac{1}{2}\right)\right)\cos\left(\frac{\pi 0}{W}\left(j+\frac{1}{2}\right)\right)\\ \text{ =}\sum_{h=0}^{H-1}\sum_{w=0}^{W-1}f_{k}\left(i,j\right) \end{array}$$

GAP is defined like this:


(3) $$\text{GAP}\left(k\right)=\frac{1}{HW}\sum_{h=0}^{H-1}\sum_{w=0}^{W-1}f_{k}\left(i,j\right)$$

which combined with (2) as follows


(4) $$Y_{0,0}^{k}=\sum_{h=0}^{H-1}\sum_{w=0}^{W-1}f_{k}\left(i,j\right)\text{ =HWGAP}\left(\text{k}\right)$$

where $Y_{0,0}^{k}$ is proportional to GAP, so GAP can be considered as a special case of the frequency components. Accordingly, only using GAP to extract features will lose information of other components, which also shows that CBAM can obtain better results by using two pooling methods for feature extraction than one pooling method. In our method, other frequency components are added to the calculation of channel attention in order to more fully obtain information from the feature maps.

**Figure 6:**
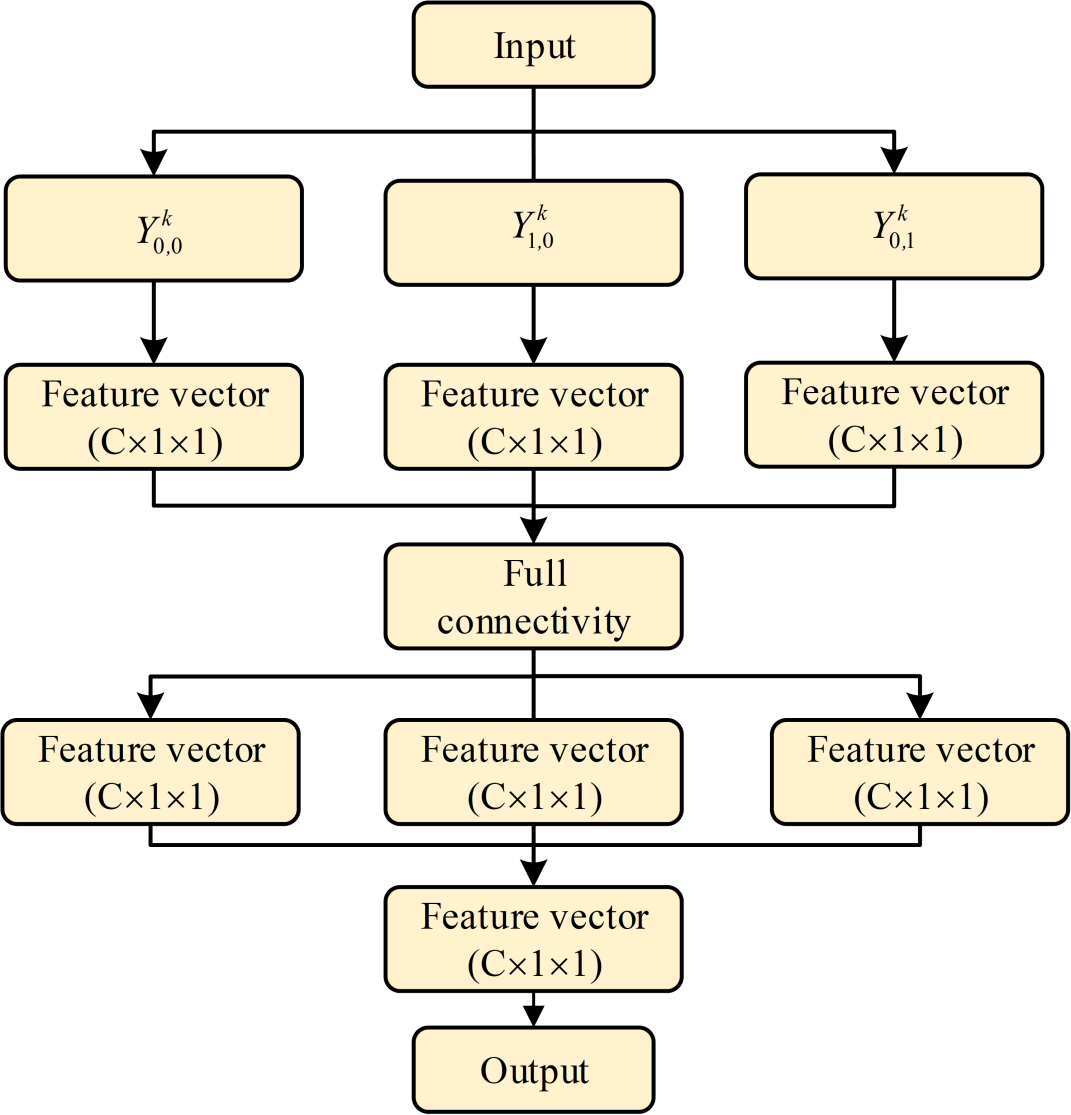
Schematic diagram of Frequency-domain Channel Attention Module.

$Y_{h,w}^{k}\in R^{C\times H\times W}$ can be calculated as a 2D-DCT of feature map $F\in R^{C\times H\times W}$ , which is composed of *CHW* frequency components. If all frequency components are included in the calculation, which will lead to high computational complexity of the network, and the network performance is not significantly improved. Xu et al. (65) proposed a method of learning in the frequency domain, analyzing frequency deviation from the frequency domain, and proving that the CNN is more sensitive to low spectral components. Accordingly, a frequency-domain channel attention model (FCAM) is proposed, which uses the low frequency components $Y_{0,0}^{k}$ , $Y_{0,1}^{k}$ , $Y_{1,1}^{k}$ of 2D-DCT, as shown in **Figure 6**.

(1) Generation of spectral components. FCAM uses discrete cosine transform to extract channel information, aiming at obtaining useful information from channel more comprehensively. FCAM performs 2D-DCT for each channel of the input. The 2D-DCT of the *k*-th channel is described in detail as follows. $Y_{0,0}^{k}\in R^{C\times 1\times 1}$ represents the spectral component at position (0,0) of 2D-DCT and is defined as:


(5) $$Y_{0,0}^{k}=\sum_{h=0}^{H-1}\sum_{w=0}^{W-1}f_{k}\left(i,j\right)\cos\left(\frac{\pi \times 0}{H}\left(i+\frac{1}{2}\right)\right)\cos\left(\frac{\pi \times 0}{W}\left(j+\frac{1}{2}\right)\right)$$

$Y_{1,0}^{k}\in R^{C\times 1\times 1}$ represents the spectral component at position (0,1) of 2D-DCT and is defined as:


(6) $$Y_{1,0}^{k}=\sum_{h=0}^{H-1}\sum_{w=0}^{W-1}f_{k}\left(i,j\right)\cos\left(\frac{\pi }{H}\left(i+\frac{1}{2}\right)\right)\cos\left(\frac{\pi \times 0}{W}\left(j+\frac{1}{2}\right)\right)$$

$Y_{0,1}^{k}\in R^{C\times 1\times 1}$ represents the spectral component at position (1,0) of 2D-DCT and is defined as:


(7) $$Y_{0,1}^{k}=\sum_{h=0}^{H-1}\sum_{w=0}^{W-1}f_{k}\left(i,j\right)\cos\left(\frac{\pi \times 0}{H}\left(i+\frac{1}{2}\right)\right)\cos\left(\frac{\pi \times 1}{W}\left(j+\frac{1}{2}\right)\right)$$

(2) Channel weights prediction. First, the feature maps $Y_{0,0\;}^{k},Y_{0,1\;}^{k},Y_{1,1\;}^{k}$ from the previous step are fed into the shared full connectivity in parallel to perform two linear mappings, the first of which is that the feature maps are linearly mapped (*W*_0_) to a vector with size *C*/*r*, followed by a rectified linear units, the second of which is that the feature maps are linearly mapped (*W*_1_) to a vector with dimension *C*, the compression rate *r* is set to 16. Second, the three feature vectors which are output by fully connected layer are added, followed by a sigmoid function. Finally, the weight coefficient *M_F_* ∈R*^C^* ^×1×1^ is obtained. The spectral attention module is defined as:


(8) $$\begin{array}{c} M_{F}=\sigma \left(MLP\left(Y_{0,0}^{k}\right)+MLP\left(Y_{0,1}^{k}\right)+MLP\left(Y_{1,0}^{k}\right)\right)\\ =\sigma \left(W_{1}\left(W_{0}Y_{0,0}^{k}\right)+W_{0}\left(W_{1}Y_{0,1}^{k}\right)+W_{0}\left(W_{1}Y_{1,0}^{k}\right)\right) \end{array}$$

where MLP is the shared fully connected layer, $W_{0}\in R^{C/r\times C}$ , $W_{1}\in R^{C\times C/r}$ , and *σ* is a sigmoid activation function.

**Figure 7:**
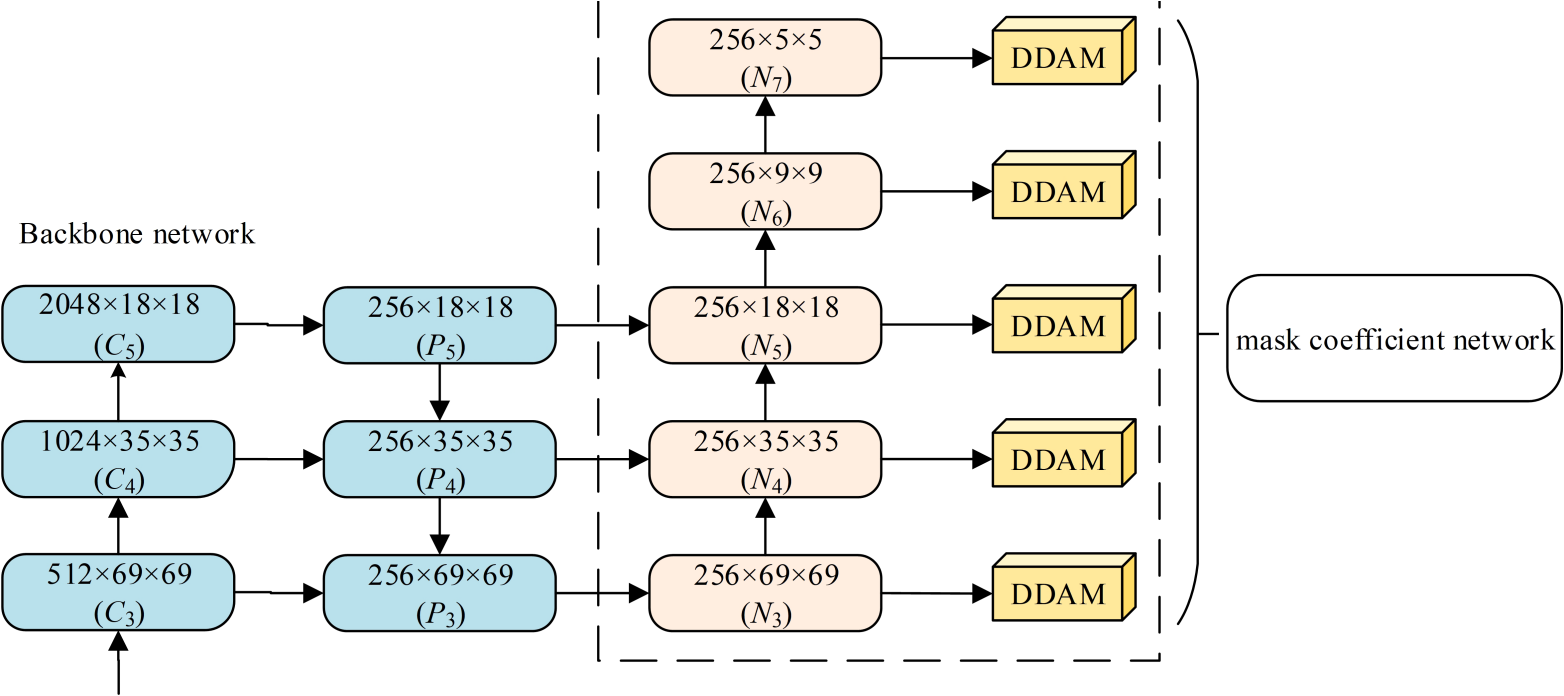
Schematic diagram of DDAM and DFFN connection.

In this paper, the improved FCAM which replaces the CAM is in series with SAM, thereby constructing a hybrid attention mechanism from frequency and spatial domains, namely dual-domain attention module (DDAM). In order to improve segmentation performance of YOLACT, our method combines the DFFN and the DDAM, as shown in **Figure 7**, the idea of which is to enable the network to recalibrate features that is given attention by itself. Given the fact that the feature layer after top-down feature fusion contains rich positioning and classification information, and the subsequent detection and classification can be more effectively recalibrated by connecting the attention module, the DDAM is placed between the DFFN and mask coefficient network. Also due to the fact that the smaller size of the feature maps output by the DFFN, connecting attention modules here cann’t increase the complexity of our method too much. Therefore, the DDAM is placed after layers *N*_3_ to *N*_7_, respectively.

## 3 Experiments and results

In this section, the dataset and preprocessing, performance evaluation metrics and hyperparameter settings used in our experiment are first introduced, and then the effectiveness of each component, a tremendous amount of ablation studies on the All-IDB1 (66) and BCCD (67) datasets are verified. Finally, experimental results of our method compared with state-of-the-art counterparts on the All-IDB1 (66), BCCD (67) and Raabin-WBC (68) datasets are reported.

### 3.1 Dataset and preprocessing

#### 3.1.1 Dataset

Due to medical image datasets usually need to be annotated by pathologists, leading to a fewer numbers of medical image datasets. Therefore, to better evaluate the performance of our method, we combine the public blood smear cell pathology image dataset ALL-IDB1 (66) from the University of Milan, Italy, and the blood smear cell pathology image dataset BCCD (67) from MIT to increase the number of blood smear images. Furthermore, to further validate our method, experiments on the Raabin-WBC dataset are conducted.

ALL-IDB1 is used for the study of white blood cells segmentation and classification. The images in the dataset were taken at a magnification of 300 to 500 of the microscope. The dataset consists of 108 images, which contains about 39000 blood elements, and each image resolution is 2592 × 1944. The BCCD Dataset includes 364 microscopic images of various white blood cells. Each image contains various blood cell components, such as white blood cells, red blood cells and platelets. The size of each image is 640 × 480 pixels. Raabin-WBC is a publicly available dataset, which contains professional annotations related to WBCs and consists of a training set (912 images) and a testing set (233 images). The size of each image is 575 × 575 pixels.

#### 3.1.2 Dataset preprocessing

In the ALL-IDB1, each image resolution is 2592 × 1944. Taking into account the limitation of the experimental equipment and directly reducing image resolution will make segmentation objects too small, which may affect the segmentation performance. Therefore, in this paper, each blood smear image is cropped with a sliding window to 512 × 512 sub-images, and the stride is set to 256. Finally, 314 images with white blood cells are obtained. In BCCD Dataset, each image resolution is 640 × 480. In order to keep the ratio of the height-to-width of each image unchanged, all images are directly zero-padded to square (640 × 640). Noting that our method uses the instance segmentation algorithm for white blood cells. However, both ALL-IDB1 and BCCD do not provide instance segmentation labels for white blood cells. Accordingly, white blood cells are annotated according to the guidance of pathologists, as shown in **Supplementary Figure 1**. In this experiment, there are 678 images in total and each image is reshaped to 550 × 550. Notably, all images are divided into a training set, a validation set and a testing set with 474, 68, 136 images respectively (i.e., a ratio of 7:1:2). Similarly, in the Raabin-WBC dataset, all images are resized into 550 × 550. Meanwhile, the Raabin-WBC dataset is divided into a training set, a validation set and a testing set with 798, 114, 233 images respectively (i.e., a ratio of 7:1:2).

### 3.2 Configuration

The segmentation method was implemented in Python on a computer equipped with two NVIDIA 1080Ti graphics cards, each of which has 11GB of memory, and a CPU of Intel Xeon E5-2630. The pytorch library served as a high-level framework, the experimental platform was based on the Ubuntu 18.04 system. The training time for our model was approximately 3.6 hours. All methods used in this experiment were compared using a set of the same hyper-parameters, ensuring the fairness of the experimental results. Regarding the selection of optimization methods, the more stable SGD method during the training process was adopted and 600 epochs were conducted to ensure our model convergence. In addition, the experience values for learning rate and batch size were chosen, and the hyper-parameter details of the segmentation model are shown in **Table 1**.

**Table 1:** Hyper-parameters of our instance segmentation model.

Model	Optimization algorithm	Learning rate	Batch size	Epoch
YOLACT-CIS	SGD	0.001	16	600

### 3.3 Performance evaluation metrics

Currently, the most widely used measures for the quantitative evaluation of image segmentation results are the following: Precision and Recall. Two criteria are defined by:


(9) $$Precision=\frac{TP}{TP+FP}$$

(10) $$Recall=\frac{TP}{TP+FN}$$

where TP and TN represent the number of the pixels that were correctly determined to be white blood cells and the background, respectively. Conversely, FP and FN represent the number of pixels that were incorrectly predicted to be white blood cells and the background, respectively. The two metrics are used to quantify the similarity between the automatically segmented white blood cell and the manually segmented white blood cell. Their values range from 0 to 1: the higher the value, the better the match.

For white blood cells instance segmentation, IoU (Intersection over Union) represents the degree of overlap between the segmentation results and the ground truth. A represents the ground truth, and B represents the segmentation result, the IoU is defined as follows:


(11) $$IoU=\frac{A\cap B}{A\cup B}$$

where the IoU threshold is 0.5, when the IoU is greater than 0.5, the segmentation result of our method is TP. The segmentation results of our method are mainly measured by mAP and AP. mAP refers to the mean AP of each category. Note that, there is only one category of detection and segmentation algorithms of white blood cells in this paper, the AP is mAP. AP_50_ is defined like this:


(12) $$AP_{50}=\int_{0}^{1}p(r)dr$$

where *p*(*r*) represents the P-R (Precision-Recall) curve, when the IoU threshold is smaller than 0.5, that is, the curve is composed of Precision and Recall, as shown in **Supplementary Figure 2**. AP is the area under the P-R curve.

In this paper, AP is the average AP_IoU_ over IoU thresholds from 0.5 to 0.95 with an interval of 0.05. In addition, there are AP_75_, FLOPs, Params and other evaluation metrics. AP_75_ means that the IoU threshold is set to 0.75, FLOPs is floating point operands, and Params is the number of the network parameters.

### 3.4 Ablation experiment

#### 3.4.1 Impact of adjustment factor *S* on network performance

Our method takes advantage of feature reuse of Ghost module to reconstruct the backbone network of the YOLACT, thereby making the network lightweight. The number of features adjustment factor *S* of Ghost module is used to control the number of feature maps generated in the first step of Ghost module, and the number of parameters and computational cost decreases with the increase of the value of *S*. In order to explore the relationship between the network complexity and the network performance, we compare the impact of different *S* values on the model performance. The experimental results are shown in **Table 2**. In this experiment, the values of *S* are set to 2, 4 and 6 respectively.

**Table 2:** The model’s performance of different ***S*** values on the ALL-IDB1 and BCCD datasets.

S	AP (%)	AP_50_ (%)	AP_75_ (%)	FLOPs (G)	Params (M)
ResNet (58)	84.86	94.81	92.03	25.66	25.56
2	85.04	94.98	92.69	14.69	13.28
4 (Ours)	**85.52**	**95.65**	**93.33**	9.19	8.16
8	83.22	93.95	91.72	**5.6**	**6.44**

Bold fonts indicate the best values in each column.

In **Table 2**, it can be seen that the network has the best performance when *S*=4, although the number of parameters and computational cost are both the smallest when *S*=8, which proves that parameter redundancy of the network results in over-fitting. But when *S*=8, the segmentation performance of the network decreased which demonstrated that excessive compression may lead to the decline of the learning capability. Accordingly, to avoid severe segmentation performance degradation, *S* is set to 4 in our experiments.

#### 3.4.2 Performance comparison of different global extraction methods in DDAM

DDAM converts spatial domain to frequency domain for global information extraction. Note that, 2D-DCT of a feature map contains many frequency components, not all of which contain useful information. Therefore, combination of different frequency components have different influences on the network performance. **Table 3** shows the performance comparison of different global feature extraction methods. $Y_{h,w}^{k}\in R^{C\times H\times W}$ is computed as a 2D-DCT of $F\in R^{C\times H\times W}$ , and then *C* × *H* × *W* frequency components are generated. In this paper, frequency components $Y_{0,0}^{k},\;Y_{0,1}^{k},\;Y_{1,0}^{k}$ and $Y_{1,1}^{k}$ are viewed as extracted global information. The following conclusions can be drawn from **Table 3**:

(1) From the comparison of two extraction methods $Y_{0,0}^{k}$ and CBAM (GMP+GAP), it indicates that the CBAM is better, which proves that it is not comprehensive to only use GAP to extract global information, and adding GMP together can supplement some missing important feature information.
(2) From the comparison of $Y_{0,0}^{k}+Y_{0,1}^{k}$ and CBAM (GMP+GAP), it can be seen that using $Y_{0,0}^{k}+Y_{0,1}^{k}$ (i.e., converting the spatial domain to the frequency domain) can improve segmentation accuracy, which demonstrates that more key information missed in the spatial domain can be extracted by $Y_{0,0}^{k}+Y_{0,1}^{k}$ . Thus, $Y_{0,1}^{k}$ is a good supplement to $Y_{0,0}^{k}$ .
(3) The feature information in $Y_{1,0}^{k}$ is somewhat different from that in $Y_{0,0}^{k}$ and $Y_{0,1}^{k}$ . Therefore, use of $Y_{1,0}^{k}$ can effectively enhance extraction of global key information, which is a supplement to other feature extraction methods
(4) From the comparison of $Y_{0,0}^{k}+Y_{0,1}^{k}+Y_{1,0}^{k}+Y_{1,1}^{k}$ and $Y_{0,0}^{k}+Y_{0,1}^{k}+Y_{1,0}^{k}$ , we can see that not all the information in the frequency components is valid, and some information may interfere with the network performance. Therefore, different combinations of the frequency components can affect the network performance to some extent.
(5) From **Table 3**, it shows that the combination of $Y_{0,0}^{k}+Y_{0,1}^{k}+Y_{1,0}^{k}$ is somewhat better than CBAM (GMP+GAP).

**Table 3:** The model’s performance of different extraction methods on the ALL-IDB1 and BCCD datasets.

Method	AP (%)	AP_50_ (%)	AP_75_ (%)
CBAM (GMP+GAP) (63)	86.12	96.53	93.87
$Y_{0,0}^{k}$	85.99	95.80	93.59
$Y_{0,0}^{k}+Y_{0,1}^{k}$	86.21	96.55	93.80
$Y_{0,0}^{k}+Y_{0,1}^{k}+Y_{1,0}^{k}$ (Ours)	**86.61**	**96.80**	**94.29**
$Y_{0,0}^{k}+Y_{0,1}^{k}+Y_{1,0}^{k}+Y_{1,1}^{k}$ .	86.31	96.61	94.01

Bold fonts indicate the best values in each column.

Consequently, the combination of $Y_{0,0}^{k}+Y_{0,1}^{k}+Y_{1,0}^{k}$ is chosen for following experiments.

#### 3.4.3 Impact of DFFN on network performance

In this section, the contribution of the DFFN to the network performance is explored. **Table 4** presents the effect comparison of DFFN and FPN methods on the network performance. The experimental results show that, compared to FPN, DFFN can capture more details, and thus effectively improving the network performance. In contrast to the FPN, the three metrics of AP, AP_50_ and AP_75_ of DFFN are 0.8%, 1.02% and 1.09% higher.

**Table 4:** The effect of feature fusion layer for model’s performance on the ALL-IDB1 and BCCD.

Method	AP (%)	AP_50_ (%)	AAP_75_ (%)
FPN (60)	86.61	96.80	94.29
DFFN	**87.41**	**97.82**	**95.38**

Bold fonts indicate the best values in each column.

#### 3.4.4 Ablation study of our method

In order to show the effect of each improvement, the following ablation study was performed. The AP, AP_50_ and AP_75_ of utilizing the adjustment factor *S*=4 achieve 0.78%, 0.89% and 1.41% gains compared with the YOLACT algorithm, respectively. When setting *S*=4 and using the DDAM, the AP, AP_50_ and AP_75_ are 1.27%, 1.20% and 1.03% higher than only employing *S*=4, respectively. When further adding the DFFN, the AP, AP_50_ and AP_75_ reach to optimal perfomance, 87.41%, 97.82% and 95.38%, respectively. As shown in **Table 5**, among all the modules the DDAM improves the performance of the AP and AP_50_ the most. Additionally, the adoption of *S*=4 contributes to the biggest improvement for the AP_75_.

**Table 5:** Ablation study of our method on the ALL-IDB and BCCD datasets.

Method	S=4	DDAM	DFFN	AP (%)	AP_50_(%)	AAP_75_(%)
1				84.86	94.81	92.03
2	✔			85.52	95.65	93.33
3	✔	✔		86.61	96.80	94.29
4	✔	✔	✔	**87.41**	**97.82**	**95.38**

Bold fonts indicate the best values in each column.

### 3.5 Performance comparison with other instance segmentation methods

In order to measure the quantitative metrics of our method, we compare our method with other instance segmentation methods. The segmentation results are shown in **Table 6**. Noting that YOLACT-CIS method obtains comprehensive improvements for nearly all metrics compared with Mask R-CNN, PointRend, MS R-CNN, SOLOv2 and YOLACT. Among them, Mask R-CNN, MS R-CNN and PointRend are two-stage instance segmentation algorithms, which generally have high segmentation accuracy, but have more network parameters and high computational cost. SOLOv2 and YOLACT are single-stage instance segmentation methods with fast segmentation speed, but segmentation accuracy is not high.

**Table 6:** Comparison with instance segmentation methods on the ALL-IDB1 and BCCD datasets.

Method	AP (%)	AP_50_ (%)	AP_75_ (%)	FLOPs (G)	Params (M)
Mask R-CNN (69)	85.72	96.92	94.64	123.48	43.75
PointRend (56)	82.39	95.90	91.60	73.26	55.48
MS R-CNN (55)	86.01	97.40	94.40	123.48	60.01
SOLOv2 (70)	63.27	89.42	75.55	\	\
YOLACT (46)	84.86	94.81	92.03	56.29	34.99
Ours	**87.41**	**97.82**	**95.38**	**39.82**	**17.59**
Ours(ALL-IDB1)	85.01	94.97	93.83	39.82	17.59
Ours(BCCD)	84.81	95.14	92.99	39.82	17.59

Bold fonts indicate the best values in each column.

In this paper, ResNet-50 is used as the backbone of all networks. As listed in **Table 6**, although the two-stage instance segmentation algorithms achieve a higher segmentation accuracy than the single-stage instance segmentation algorithms. The AP, AP_50_ and AP_75_ of our method are 1.40-24.14%, 0.42-8.40% and 0.74-19.83% higher than those of other algorithms, respectively. In the meantime, our method significantly reduces the number of parameters and FLOPs. In the meantime, our method significantly reduces the number of parameters and FLOPs, which is 50.3% and 70.7% of YOLACT respectively. Furthermore, the above-mentioned six methods in **Table 6** are also validated on the Raabin-WBC dataset. In **Supplementary Table 1**, it is easily observed that our method outperforms the other methods, demonstrating that the proposed method attains superior performance in terms of AP, AP50, and AP75. Besides, **Supplementary Table 2** shows computation time comparison of instance segmentation methods. Our method is the fastest among all methods.

**Figure 8:**
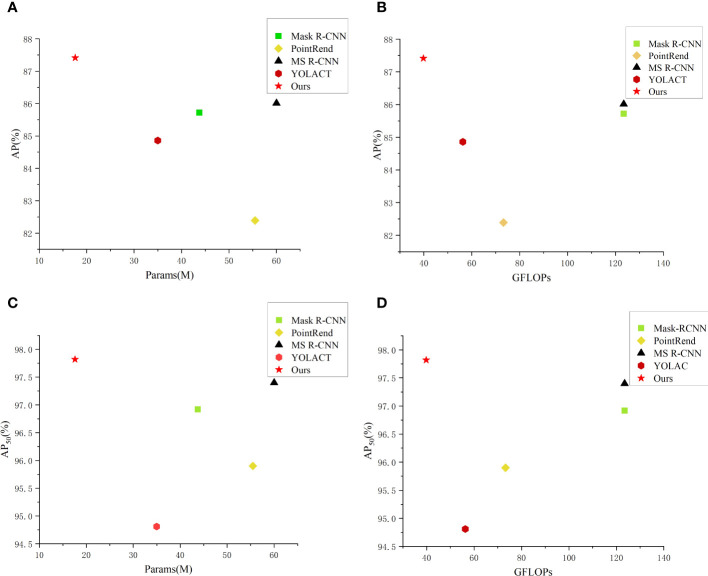
Comparison of parameters and performance of different instance segmentation networks. **(A)** AP-Params scatter plot; **(B)** AP-GFLOPs scatter plot; **(C)** AP_50_-Params scatter plot; **(D)** AP_50_-GFLOPs scatter plot.

Moreover, to more intuitively compare performance of the aforementioned methods, we have constructed scatter plots of the number of the network parameters and FLOPs. From **Figure 8**, it can be seen that our method achieves best results in the segmentation accuracy and the network lightweight compared with other methods.

**Figure 9:**
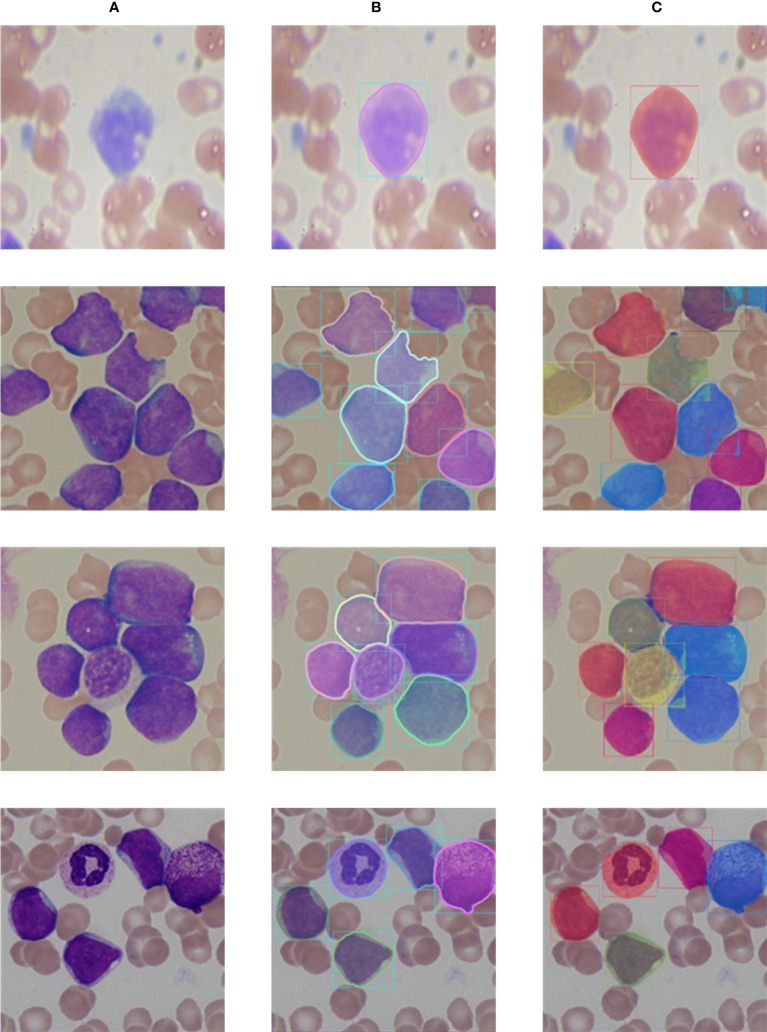
Comparison of labels and prediction results. **(A)** Input images; **(B)** Labels; **(C)** Prediction results.

The visualization segmentation results of our method are shown in **Figure 9**. It can be seen that each white blood cell in the blood smear images can be accurately detected, and our method can more completely segment the white blood cells. It is worth noting that for white blood cells with overlapping and irregular edge, our method still performs well, and overlapping and adhesive contours between cells can be completely segmented. The visualization segmentation results further indicate our method has good segmentation performance.

## 4 Discussion

First, our method is significantly better than the current state-of-the-art single-stage methods in terms of both the number of parameters and FLOPs, and our method has the best performance among all methods. However, the performance of our method is still lower than the two-stage instance segmentation algorithms. Second, there are still some equipment with insufficient performance, so the lightweight network research needs to be further explored. In order to make white blood cells segmentation methods more practical, in future work, how to design a more lightweight network model while ensuring a good accuracy will become an important problem.

Because a large number of annotated data are required for cell segmentation in deep learning methods, the training of our method has used conventional data augmentation methods. The effect of data augmentation on the ALL-IDB1 and BCCD datasets is analyzed in **Supplementary Table 3**. In the future, semi-supervised learning and data distillation can be used to reduce the need for a large number of annotated data. Also, the generative adversarial network can also be used to augment datasets.

## 5 Conclusion

In summary, motivated by the instance segmentation network YOLACT, according to features of cell in cytopathological images, we propose an instance segmentation model named YOLACT-CIS to segment white blood cells in cytopathological images. First, the Ghost module has been used to make the structure of the backbone network lightweight, aiming at reducing the number of network parameters and computational cost. Second, a novel DFFN is proposed. Specifically, a bottom-up path has been added to the fusion layer of FPN to improve the capability of obtaining detailed feature information. Finally, the DDAM is proposed to extract global features from both frequency and spatial domains simultaneously, so as to enhance the capability to extract features. Adequate experimental results proved that our proposed method can further lighten the network structure while achieving competitive white blood cells segmentation performance compared with other state-of-the-arts. In the future, we will validate our method in more medical image segmentation scenarios.

## Data availability statement

The original contributions presented in the study are included in the article/**Supplementary Material**, further inquiries can be directed to the corresponding author.

## Author contributions

Conceptualization: YL and CF. Methodology: YL, YW, HS, and YZ. Data acquisition: WG and YZ. Investigation: CF and WG. Writing- original draft preparation: YL. Writing-review and editing: YL and HJ. Supervision:YW and HJ. Funding acquisition: YL. All authors contributed to the article and approved the submitted version.
