# Supplementary figures and images for: A lightweight network based on dual-stream feature fusion and dual-domain attention for white blood cells segmentation
Source: Front Oncol. 2023 Sep 4;13:1223353. doi: 10.3389/fonc.2023.1223353 (PMC10507331; doi:10.3389/fonc.2023.1223353)

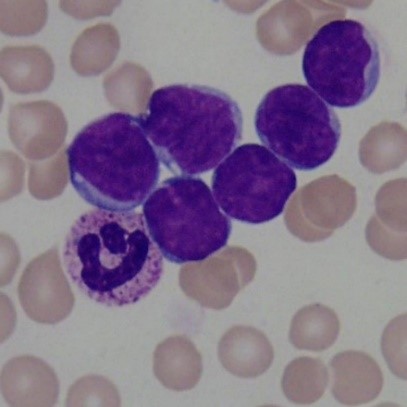

Supplement: Supplementary file 1 [file Image_1.jpeg]

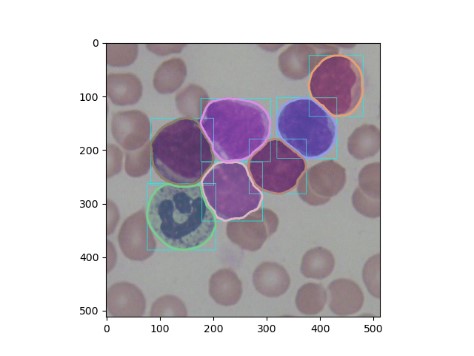

Supplement: Supplementary file 2 [file Image_2.jpeg]
