# Supplementary material for: A lightweight network based on dual-stream feature fusion and dual-domain attention for white blood cells segmentation
Source: Front Oncol. 2023 Sep 4;13:1223353. doi: 10.3389/fonc.2023.1223353 (PMC10507331; doi:10.3389/fonc.2023.1223353)
Supplement: Supplementary file 3 [file Table_1.docx]

Supplementary Table 1. Comparison with instance segmentation methods on the Raabin-WBC dataset.

| Method | AP (%) | AP_50_ (%) | AP_75_ (%) |
| --- | --- | --- | --- |
| Mask R-CNN ^[69]^ | 80.79 | 96.14 | 90.97 |
| PointRend ^[56]^ | 80.72 | 96.75 | 95.72 |
| MS R-CNN ^[55]^ | 81.37 | 98.55 | 91.18 |
| SOLOv2 ^[70]^ | 68.37 | 92.82 | 77.66 |
| YOLACT^[46]^ | 77.49 | 98.11 | 95.13 |
| Ours | **90.27** | **98.91** | **96.41** |
