# Supplementary material for: A lightweight network based on dual-stream feature fusion and dual-domain attention for white blood cells segmentation
Source: Front Oncol. 2023 Sep 4;13:1223353. doi: 10.3389/fonc.2023.1223353 (PMC10507331; doi:10.3389/fonc.2023.1223353)
Supplement: Supplementary file 4 [file Table_2.docx]

Supplementary table 2. Computation time comparison of instance segmentation methods on the ALL-IDB1 and BCCD datasets.

| Method | FPS | Time (s) |
| --- | --- | --- |
| Mask R-CNN ^[69]^ | 11.5 | 87.0 |
| PointRend ^[56]^ | 7.1 | 140.8 |
| MS R-CNN ^[55]^ | 11.5 | 87.0 |
| SOLOv2 ^[70]^ | 45.0 | 22.2 |
| YOLACT^[46]^ | 42.0 | 23.8 |
| Ours | **46.3** | **21.6** |
