# Supplementary material for: A lightweight network based on dual-stream feature fusion and dual-domain attention for white blood cells segmentation
Source: Front Oncol. 2023 Sep 4;13:1223353. doi: 10.3389/fonc.2023.1223353 (PMC10507331; doi:10.3389/fonc.2023.1223353)
Supplement: Supplementary file 5 [file Table_3.docx]

Supplementary Table 3. The effect of data augmentation on the ALL-IDB1 and BCCD datasets.

|  | AP (%) | AP_50_ (%) | AP_75_ (%) |
| --- | --- | --- | --- |
| without-aug | 85.17 | 94.92 | 92.19 |
| with-aug | **87.41** | **97.82** | **95.38** |
